# Enhancement of Solubility of Lamotrigine by Solid Dispersion and Development of Orally Disintegrating Tablets Using 3^2^ Full Factorial Design

**DOI:** 10.1155/2015/828453

**Published:** 2015-10-27

**Authors:** Jatinderpal Singh, Rajeev Garg, Ghanshyam Das Gupta

**Affiliations:** Department of Pharmaceutics, ASBASJSM College of Pharmacy, BELA, Ropar, Punjab 140111, India

## Abstract

Present investigation deals with the preparation and evaluation of orally disintegrating tablets (ODTs) of lamotrigine using *β*-cyclodextrin and PVP-K30 as polymers for the preparation of solid dispersion which help in enhancement of aqueous solubility of this BCS CLASS-II drug and sodium starch glycolate (SSG) and crospovidone as a superdisintegrating agent, to reduce disintegration time. The ODTs were prepared by direct compression method. Nine formulations were developed with different ratios of superdisintegrating agents. All the formulations were evaluated for disintegration time, weight variation, hardness, friability, drug content uniformity, wetting time, and *in vitro* drug release study. *In vitro* drug release study was performed using United States Pharmacopoeia (USP) type 2 dissolution test apparatus employing paddle stirrer at 50 rpm using 900 mL of 0.1 N HCl maintained at 37°C ± 0.5°C as the dissolution medium. On the basis of evaluation parameters formulations were prepared using *β*-CD 1 : 1 solid dispersion. Then 3^2^ full factorial design was applied using SSG and crospovidone in different ratios suggested by using design expert 8.0.7.1 and optimized formulation was prepared using amount of SSG and crospovidone as suggested by the software. The optimized formulation prepared had disintegrating time of 15 s, wetting time of 24 s, and % friability of 0.55.

## 1. Introduction

Convenience of administration and patient compliance are gaining significant importance in the design of dosage forms. Recently more stress is laid down on the development of organoleptically elegant and patient friendly drug delivery systems [1]. Although various novel and advanced drug delivery systems have been introduced for therapeutic use, the popularity of oral dosage forms has not been eclipsed [2]. The oral route remains the preferred route of drug administration due to its convenience, good patient compliance, and low medicine production costs. To meet these medical needs, formulators have devoted considerable efforts to develop an innovative dosage form known as orally disintegrating tablet (ODT) [3]. A major claim of the some ODTs is increased bioavailability compared to traditional tablets [4]. One of the major challenges to drug development today is poor solubility; as estimated most of the developed drugs are poorly soluble or insoluble in water.

Dysphagia, or difficulty in swallowing, is common among all age groups. According to a study by Sastry et al. [5], dysphagia is common in about 35% of the general population.

Elderly and pediatric patients and traveling patients who may not have ready access to water generally need easy swallowing dosage forms. Study showed that an estimated 50% of the population suffers from this problem [6].

Further, drugs exhibiting satisfactory absorption from the oral mucosa or intended for immediate pharmacological action can be advantageously formulated in these dosage forms. Therefore, research on developing orally disintegrating systems has been aimed at investigating different excipients as well as techniques to meet these challenges.

Taste masking of active ingredients becomes essential in these systems because the drug is completely released in the mouth. It is important that freeze-dried and effervescent disintegrating systems rapidly disintegrate in contact with fluids; they do not generally exhibit the required mechanical strength. In the same way, the candy process cannot be used for thermolabile drugs. It is also accountable that these techniques differ in their methodologies and the ODTs formed vary in various properties such as mechanical strength of tablets, taste and mouth feel, and swallowability, drug dissolution in saliva, bioavailability, and stability [7].

Lamotrigine, an antiepileptic drug (AED) of the phenyltriazine class, is chemically unrelated to existing antiepileptic drugs. For epilepsy it is used to treat partial seizures, primary and secondary tonic-clonic seizures, and seizures associated with Lennox-Gastaut syndrome. It is also used in the treatment of depression and bipolar disorder [8]. Lamotrigine has relatively few side-effects and does not require blood monitoring in monotherapy [9]. Lamotrigine is thought to exert its anticonvulsant effect by stabilizing presynaptic neuronal membranes; it inhibits sodium currents by selectively binding to the inactivated state of the sodium channel and subsequently suppresses the release of the excitatory amino acid, glutamate.

Lamotrigine was selected for the present work because it is BCS class II drug and has solubility problems. BCS class II (i.e., less water soluble) drugs require innovative approaches to reach a sufficiently high bioavailability when administered by oral route. Poorly water soluble drugs can exhibit a number of negative clinical effects including potentially serious issues of interpatient variability and subsequent erratic absorption. Lamotrigine is very slightly soluble in water (0.17 mg/mL at 25°C) and slightly soluble in 0.1 M HCl (4.1 mg/mL at 25°C), having plasma half-life of 24 to 35 hours [10]. Secondly it has bitter taste, which decreases patient compliance when taken orally; both these problems were eliminated by preparing its solid dispersion with *β*-CD. *β*-CD make inclusion complex with drug and bitter taste of the drug can be masked [11]. By taking into account all these aspects it was planned to formulate orally disintegrating tablets containing solid dispersion of lamotrigine because orally disintegrating systems become more popular than other oral drug delivery systems due to the highest component of compliance they offered to the patients, especially to the geriatrics and pediatrics. In addition, patients suffering from dysphagia, motion sickness, repeated emesis, and mental disorders prefer these medications because they cannot swallow large quantity of water [12].

## 2. Materials and Method

Lamotrigine was obtained as a gift from IPCA laboratories LTD, kandivali, Mumbai. and sodium starch glycolate, mannitol, sodium saccharin, and crospovidone were received as gift samples from Signet Chemicals, Mumbai, India. *β*-cyclodextrin was purchased from Himedia Laboratories Pvt ltd. Magnesium stearate, hydrochloric acid, polyvinyl pyrrolidone K30 (PVP K30), Avicel PH102, and all other chemicals used were of analytical grade.

Solid dispersion was prepared with PVP-K30 and *β*-CD using kneading method. ODT tablets were prepared by using 3^2^ full factorial design using design expert trial 8.0.7.0 by direct compression. One-way analysis of variance (ANOVA) was adopted to find out the significance of* in vitro* drug release data at 5% level of significance (*p* < 0.05) [13].

## 3. Solid Dispersion Preparation

For the enhancement of solubility and dissolution of lamotrigine, solid dispersion and inclusion complexes were prepared using PVPK30 and *β*-cyclodextrin, respectively. Kneading method was used to prepare solid dispersion of lamotrigine.  Table 1 depicts the composition for preparing solid dispersion of lamotrigine with polyvinyl pyrrolidone K30 and  *β*-CD in various ratios. Lamotrigine and polymers were weighed according to different weighted ratios. The physical mixtures were wetted with water-methanol (1 : 9) mixture and kneaded thoroughly for 30 min in a glass mortar. The paste formed was dried under vacuum for 24 h. Dried powder was passed through sieve no. 60 and stored in a desiccator until further evaluation [14].  Table 2 represents the drug content and solubilities of various solid dispersion.

## 4. Tablet Preparation

All tablets containing magnesium stearate as lubricant were prepared by direct compression. The respective powders were weighed according to full factorial design and (drug : *β*-CD solid dispersion (1 : 1) (weight per weight), SSG, crospovidone, mannitol, Avicel PH-102, sodium saccharin (as sweetening agent), magnesium stearate, and other excipients listed in Table 3) were blended thoroughly with a mortar and pestle. The amount of both superdisintegrants was varied in the range of 1–3%. Then the mixture was weighed and fed manually into the die of an instrumented single-punch tablet machine (Cadmach, Ahmedabad) to produce tablets using flat-faced punches. The hardness of the tablets was kept constant and was measured with a hardness tester. The various pre- and postcompression parameters of blend and tablets, respectively, are shown in Tables 4 and 5.

## 5. Full Factorial Design

A 3^2^ randomized full factorial design was adopted to optimize the variables [15]. In this design the experimental trials were performed at all 9 possible combinations. The amounts of superdisintegrants, *X*_1_ (crospovidone) and *X*_2_ (sodium starch glycolate), were selected as independent variables. The disintegration time (DT) and percent friability (%*F*) and wetting time (WT) were selected as dependent variables. Low (−1), medium (0), and high (+1) are the values of *X*_1_ (crospovidone) and *X*_2_ (sodium starch glycolate), respectively. All the possible batches of factorial design are shown in Table 3.

After inserting the values of dependent variables in the design expert software the goals were set as shown in Table 6. The concentration of SSG and crospovidone was kept within range, disintegration time (DT) was targeted 15 s, wetting time (WT) was kept in range of 11–32 s, and friability was minimized. The solution was suggested for this goal by the software according to which optimized batch was prepared which had close relation with the values of dependent variables as suggested by the software.

## 6. Evaluation Parameters

### 6.1. Determination of Drug Content

Drug content was calculated by dissolving physical mixtures and solid dispersion equivalent to 10 mg LAMO in 10 mL of methanol, filtered using Whatman filter paper (number 41), suitably diluted with 0.1 N HCL, and analyzed by using UV spectrophotometer against 0.1 N HCL as blank.

### 6.2. Determination of Solubility

Pure lamotrigine and solid dispersion equivalent to 10 mg of lamotrigine were added to 10 mL of 0.1 N HCL in a 10 mL volumetric flask. The volumetric flasks were capped properly and shaken at 37°C in a temperature controlled water bath (shaking water bath) for 48 h. Resultant samples containing undissolved solid dispersion suspended in the volumetric flask were filtered through Whatman filter paper (number 41), suitably diluted with 0.1 N HCL, and analyzed by UV spectrophotometer at 267.5 nm.

### 6.3. *In Vitro* Drug Release

Accurately weighed solid dispersion equivalent to 10 mg of lamotrigine was added to 900 mL of dissolution medium, that is, 0.1 N HCl in USP II Paddle type apparatus, and stirred at a speed of 50 rpm at 37 ± 0.50°C. 10 mL aliquots were withdrawn at 2, 4, 6, 8, 10, 15, 20, 25, and 30 minutes and replaced by 10 mL of fresh dissolution media. The collected samples were analyzed after filtration and dilution at 267.5 nm using UV-visible spectrophotometer against the blank. Drug release studies were carried out in triplicate. The dissolution studies of pure lamotrigine are performed similarly. The release profile data was analyzed for cumulative percent drug released at different time intervals and for dissolution efficiency at 6 and 10 minutes.

### 6.4. Bulk Density

Bulk density is defined as the mass of powder divided by the bulk volume and is expressed as g/cm^3^. Apparent bulk density (*ρ*_*b*_) was determined by pouring the blend into a graduated cylinder. The bulk volume (*V*_*b*_) and weight of powder (*M*) were determined. The bulk density was calculated using the the following formula:

(1) $$\begin{array}{c} \rho_{b}=\frac{M}{V_{b}}. \end{array}$$

### 6.5. Tapped Density

Tapped density (*ρ*_*t*_) can be defined as mass of blend in the measuring cylinder divided by its tapped volume. The measuring cylinder containing a known mass of blend was tapped 100 times using tapped density apparatus. The minimum volume (*V*_*t*_) occupied in the cylinder and the weight (*M*) of the blend were measured. The tapped density was calculated using the following formula:

(2) $$\begin{array}{c} \rho_{t}=\frac{M}{V_{t}}. \end{array}$$

### 6.6. Compressibility Index [16]

The parameter is used to evaluate flowability of a powder by comparing the bulk density and tapped density of a powder using the following formula, known as Carr's compressibility index (%):

(3) Carr's  Index=Tapped  density−Bulk  densityTapped  density×100.

### 6.7. Hausner's Ratio

Hausner ratio (HR) is an indirect index of ease of powder flow. It is calculated by the following formula:

(4) $$\begin{array}{c} \text{HR}=\frac{\rho_{t}}{\rho_{b}}, \end{array}$$

where *ρ*_*t*_ is tapped density and *ρ*_*b*_ is bulk density.

A Hausner ratio of less than 1.25 (equivalent to 20% Carr) indicates good flow, while that of greater than 1.5 (equivalent to 33% Carr) indicates poor flow. A Hausner ratio between 1.25 and 1.5 glidants can be added to improve flow.

### 6.8. Angle of Repose

Angle of Repose was determined using funnel method. The blend was poured through a funnel that can be elevated vertically until a specified cone height (*h*) was obtained. Radius of the heap (*r*) was measured and angle of repose (*θ*) was calculated using the following formula:

(5) $$\begin{array}{c} \tan\theta =\frac{h}{r};\;\text{therefore; }\theta =\tan^{-1}\left(\frac{h}{r}\right). \end{array}$$

### 6.9. Tablet Thickness

Tablet thickness is an important characteristic in reproducing appearance and also in counting by suing filling equipment. Some filling equipment utilizes the uniform thickness of the tablets as a counting mechanism. Ten tablets were taken and their thickness was recorded using micrometer (Mitutoyo, Japan).

### 6.10. Uniformity of Weight

As per IP, twenty tablets were taken and weighed individually and collectively using digital balance. The average weight of one tablet was calculated. The weight variation test would be satisfactory method of determining the drug content uniformity [17].

### 6.11. Tablet Hardness

It can be defined as the force required per unit area to break the tablet. The resistance of the tablet to chipping, abrasion, or breakage under conditions of storage transformation and handling before usage depends on its hardness. Hardness of the tablets was determined by using Monsanto hardness tester [18].

### 6.12. Friability

Friability of the tablets was determined using Roche friabilator. This device subjects the tablets to the combined effect of abrasions and shock in a plastic chamber revolving at 25 rpm and dropping the tablets at a height of 6 inches in each revolution. Preweighed sample of tablets was placed in the friabilator and subjected to 25 rpm for 4 minutes (100 revolutions). Tablets were dusted using a soft muslin cloth and reweighed. The friability (%*F*) is determined by the following formula [19]:

(6) $$\begin{array}{c} \%F=\left(\;1-\frac{W_{0}}{W}\;\right)\times 100, \end{array}$$

where *W*_0_ is initial weight of the tablets before the test and *W* is the weight of the tablets after test.

### 6.13. Disintegration Test

Disintegration of orally dissolving tablets is achieved in the mouth owing to the action of saliva; however amount of saliva in the mouth is limited and no tablet disintegration test for mouth dissolving tablets was found in USP and IP to simulate* in vivo* conditions. A modified method was used to determine disintegration time of the tablets. A cylindrical vessel was used in which 10 meshscreen was placed in such way that only 2 mL of disintegrating or dissolution medium would be placed below the sieve. To determine disintegration time, 6 mL of phosphate buffer (pH 6.8) was placed inside the vessel in such way that 2 mL of the media was above the sieve and 4 mL of the media was below the sieve. Tablet was placed on the sieve and the whole assembly was then placed on a shaker. The time, at which all the particles pass through the sieve, was taken as a disintegration time of the tablet.

### 6.14. Wetting Time

The method was followed to measure tablet wetting time. A piece of tissue paper (12 cm × 10.75 cm) folded twice was placed in a small Petri dish (Internal Diameter = 65 cm) containing 10 mL of 0.1 N HCl. A tablet was put on the paper, and the time for the complete wetting was measured.

### 6.15. *In Vitro* Dispersion Time

*In vitro* dispersion time was measured by dropping a tablet in a glass cylinder containing 6 mL of 0.1 N HCL. Three tablets from each formulation were randomly selected and* in vitro* dispersion time was performed.

## 7. Results and Discussion

**Figure 1:**
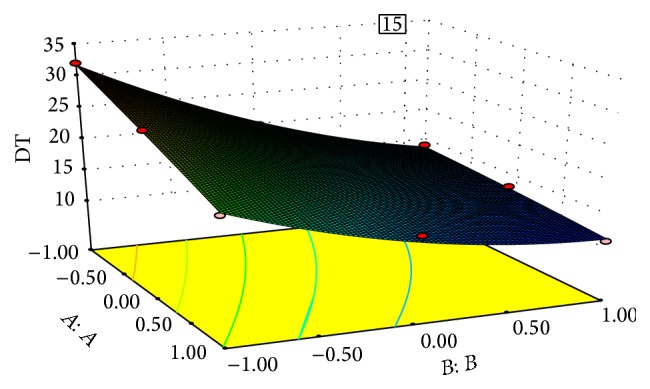
Response surface plot of disintegrating time (DT).

Solid dispersion (SD) of lamotrigine with betacyclodextrin and PVP-K30 (1 : 1 to 1 : 3) was prepared by kneading technique; the prepared solid dispersion was evaluated for percent drug content, solubility studies, and* in vitro* drug release as shown in Figure 1. The compositions of various formulations of solid dispersion are shown in Table 1.

The drug content of solid dispersion (LP1–LB3) was found to be from 97.8 to 99.9, which is found to be within the range of ±5% of the theoretical claim (Table 2), which showed the uniformity and reproducibility of the obtained method. The saturation solubility of pure drug and solid dispersion was found to be 0.16 mg/mL and 0.83 mg/mL as shown in Table 2. It was observed that the saturation solubility of drug was increased by 4-5-folds by converting the drug into solid dispersion, due to change in physical state of lamotrigine from crystalline to amorphous state.

For tablets prepared using superdisintegrants, the bulk density of blends varied between 0.598– and 0.678 g/cc. The tapped density was found in the range of 0.782–0.672 g/cc. By using these two density data, Hausner's ratio and compressibility index were calculated. Blends having value of compressibility index less than 16% were considered as free flowing ones. The values for compressibility index were found between 11.362 and 12.395%. The powder blends of all formulation had Hausner's ratio of less than 1.25 indicating good flow characteristics. The flowability of the powder was also evidenced by the angle of repose. The angle of repose below 30° range indicated good to excellent flow properties of powder. The lower the friction occurring within the mass, the better the flow rate. The angle of repose was found to be in range (Table 4).

The mixed blends were then compressed using single-punch tablet machine. After compression of powder, the tablets obtained were evaluated for their organoleptic (color and odor), physical (size, shape, and texture), and quality control parameters (diameter, thickness, hardness, friability, disintegration time, and wetting time). All the formulations were white in color and flat in shape with smooth surface not having any defects. The average weight of the prepared tablets was found between 151.8 and 146.5 mg. The thickness of the tablets varied between 3.175 and 3.025 mm. The friability of all the formulations was found to be less than 1.0%. The hardness of tablets varied from 2.7 to 3.1 kg/cm^2^ (Table 5).

Superdisintegrants were incorporated in the formulations to facilitate quicker disintegration of the tablet as soon as it contacts the saliva in the mouth. These disintegrants act by drawing water into the tablet owing to the wicking or capillary action leading to swelling and breakup of the tablet. In the formulation of ODTs, two superdisintegrants (sodium starch glycolate and crospovidone) were tested in different concentrations. The disintegration process of the tablet was fully dependable on nature and concentration of superdisintegrant used.

### 7.1. Full Factorial Design

A 3^2^ randomized full factorial design was used in the present study to study the effect of concentration of 2 superdisintegrants as factors on the disintegration property, wetting time, and percent friability. In this design, 3 factors were evaluated, each at 3 levels, and experimental trials were performed at all 9 possible combinations. The amounts of SSG (sodium starch glycolate) (*X*_1_) and the amount of crospovidone (*X*_2_) were selected as independent variables. The disintegration time, percentage friability and wetting time were selected as dependent variables. A statistical model incorporating interactive and polynomial terms was used to evaluate the responses:

(7) $$\begin{array}{c} Y=b_{0}+b_{1}X_{1}+b_{2}X_{2}+b_{12}X_{1}X_{2}+b_{11}X_{1}^{2}+b_{22}X_{2}^{2}, \end{array}$$

where *Y* is the dependent variable, *b*_0_ is the arithmetic mean response of the 9 runs, and *b*_*i*_ is the estimated coefficient for the factor *X*_*i*_. The main effects (*X*_1_ and *X*_2_) represent the average result of changing 1 factor at a time from its low to high value. The interaction terms (*X*_1_ *X*_2_) show how the response changes when 2 factors were simultaneously changed. The polynomial terms (*X*_1_^2^ and *X*_2_^2^) were included to investigate nonlinearity.

The disintegration time, wetting time, and percentage friability for the SSG and crospovidone combination (batches ODT1 to ODT9) showed a wide variation (i.e., 11–32 s, 21–40 s, and 0.52–0.69, resp.). The results were shown in Table 5. The data clearly indicated that the disintegration time, wetting time, and percentage friability are strongly dependent on the selected independent variables. The fitted equation relating the responses disintegration time, percentage friability, and wetting time to the transformed factor is shown in Table 6. The polynomial equations (see (8)) can be used to draw conclusions after considering the magnitude of coefficient and the mathematical sign it carries (i.e., positive or negative).  Table 7 showed the results of the analysis of variance (ANOVA), which was used to generate mathematical models:

(8) DT=17.44−3.17X1−7.00X2+2.00X1X2−0.17X1X1+2.33X2X2,WT=25.78−2.83X1−6.67X2+1.75X1X2−0.83X1X1+2.33X2X2,%F=0.56−0.017X1−0.060X2+0.020X1X2+1.00X1X1−0.030X2X2.

The high values of correlation coefficient for disintegration time, % friability, and wetting time indicate a good fit, that is, good agreement between the dependent and independent variables. The equations may be used to obtain estimates of the response as a small error of variance was noticed in the replicates. The *F* value in the ANOVA table was the ratio of model mean square (MS) to the appropriate error (i.e., residual) mean square. The larger the ratio is, the larger the *F* value is and the more likely that the variance contributed by the model was significantly larger than random error. If the *F* ratio, the ratio of variances, lies near the tail of the 〈*F*〉 distribution, then the probability of a larger *F* is small and the variance ratio was judged to be significant. Usually, a probability less than 0.05 is considered significant. Values of “*p*” less than 0.0500 indicate that model terms are significant. In this case the models generated for disintegration time, percent friability, and wetting time were found significant. As there were no insignificant terms, model reduction is not required. The *F* distribution is dependent on the degrees of freedom 〈DF〉 for the variance in the numerator and the 〈DF〉 of the variance in the denominator of the *F* ratio. The model *F* value of 128.63 for disintegration time, 133.28 for wetting time, and 48.36 for friability and high *R* ^2^ values suggested that these models are significant.

### 7.2. Effect of Independent Variable on Dependent Variable

**Figure 2 fig2:**
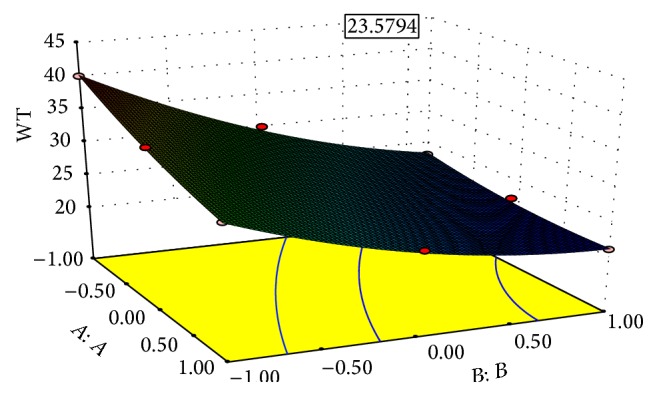
Response surface graph for wetting time (WT).

**Figure 3 fig3:**
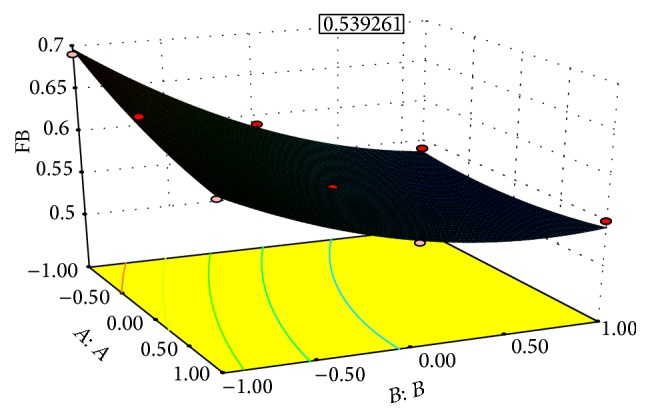
Response surface graph for % friability (FB).

The results of multiple linear regression analysis revealed that, on increasing the concentration of both the sodium starch glycolate and the crospovidone, a decrease in disintegration time was observed; both coefficients *b*_1_ and *b*_2_ bear a negative sign. Decrease in disintegration time is more significant in case of crospovidone than sodium starch glycolate. By increasing the concentration of crospovidone disintegration time increases more rapidly than in case of sodium starch glycolate. It is obvious that, in the presence of higher percentage of superdisintegrant crospovidone, wicking is facilitated. In case of percent friability, conclusions can be drawn considering the magnitude of the coefficient and the mathematical sign (positive or negative) it carries. The increase in the concentration of crospovidone results in decreased friability values. Also crospovidone produces mechanically stronger tablets than that of sodium starch glycolate, so with the increase in concentration of crospovidone friability decreases. These results were also shown in the response surface plots (Figures 1–4).

The optimization of the ODT was decided to target disintegration time 15 s and percent friability is minimized and wetting time is within range. The optimized concentration was obtained by software as clear in the surface response prediction curves. A checkpoint batch was prepared at *X*_1_ = −0.36 level and *X*_2_ = 0.56 level. From the full model, it was expected that the friability value of the checkpoint batch should be 0.52, the value of disintegration time should be 15.00 s, and the value of wetting time should be 23.58 s. The obtained results were found as expected. Thus, we can conclude that the statistical model was mathematically valid.

## 8. Conclusion

From all of the solid dispersion prepared it was clear that solubility of drug increases with increase in the amount of both carriers but PVP-K30 showed more increase in solubility than *β*-CD and trial batches of ODTs were prepared with selected solid dispersion of both carriers, that is, PVP K30 and *β*-CD; the tablets made with LP3 showed high values of disintegration time because in higher concentrations it acts as binder and therefore increases the disintegration time so PVP K30 solid dispersion was not used for the preparation of ODT, thus *β*-CD was used for the preparation of solid dispersion as it showed more release in first 5 min. than other solid dispersion by incorporating lesser carrier than others which also helps in keeping the weight of the final dosage form within range. Secondly *β*-CD makes inclusion complex with the drug which masks the bitter taste of the drug simultaneously.

From the evaluation of the parameters of the various batches of the ODTs it was clear that both superdisintegrants decrease the disintegration time but crospovidone showed more stronger affect than SSG; secondly it produced mechanically harder tablets than SSG. Crospovidone showed its action by swelling and wicking action.

As crospovidone facilitates wicking effect, it also reduces the wetting time more effectively than SSG. So it was concluded that optimization helps in selecting the appropriate amount of dependent variables to achieve the required goal.

## Figures and Tables

**Figure 4:**
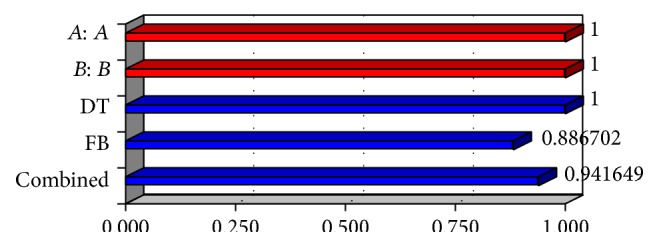
Response surface graph for desirability.

**Table 1:** Composition of solid dispersions.

Formulation number	Drug : carrier
LP1	1 : 1
LP2	1 : 2
LP3	1 : 3
LB1	1 : 1
LB2	1 : 2
LB3	1 : 3

LP = lamotrigine: PVP-K30 and LB = lamotrigine: *β*-CD.

**Table 2:** Drug content and solubility of solid dispersions.

Formulation number	% drug content	Solubility (mg/mL)
Pure drug	—	0.16 ± 0.001
LP1	98.3 ± 1.221	0.40 ± 0.013
LP2	98.7 ± 1.880	0.55 ± 0.003
LP3	99.1 ± 1.551	0.83 ± 0.003
LB1	99.9 ± 1.550	0.52 ± 0.002
LB2	98.4 ± 1.253	0.61 ± 0.001
LB3	97.8 ± 1.503	0.77 ± 0.001

**Table 3:** Composition of drug, polymers, and different excipients.

Ingredients	ODT1	ODT2	ODT3	ODT4	ODT5	ODT6	ODT7	ODT8	ODT9
LB1	50	50	50	50	50	50	50	50	50
SSG	1.5	3	4.5	1.5	3	4.5	1.5	3	4.5
Crospovidone	1.5	1.5	1.5	3	3	3	4.5	4.5	4.5
Mannitol	20	20	20	20	20	20	20	20	20
Avicel pH 102	71.5	70	68.5	70	68.5	67	68.5	67	65.5
Sodium saccharin	0.5	0.5	0.5	0.5	0.5	0.5	0.5	0.5	0.5
Mg stearate	5	5	5	5	5	5	5	5	5

**Table 4:** Precompression parameters of blends SD ± (*n* = 6).

Parameters formulation	Bulk density (g/cc)	Tapped density (g/cc)	Hausner's Ratio	Compressibility index (%)	Angle of repose (°)
ODT1	0.598 ± 0.007	0.782 ± 0.006	1.149 ± 0.014	12.995 ± 1.105	22 ± 3.023
ODT2	0.590 ± 0.010	0.672 ± 0.006	1.138 ± 0.027	12.107 ± 2.119	24 ± 1.564
ODT3	0.609 ± 0.016	0.702 ± 0.011	1.146 ± 0.025	12.738 ± 1.958	23 ± 2.654
ODT4	0.669 ± 0.024	0.757 ± 0.025	1.131 ± 0.015	11.599 ± 1.213	25 ± 1.589
ODT5	0.598 ± 0.014	0.680 ± 0.018	1.137 ± 0.024	12.078 ± 1.916	28 ± 1.852
ODT6	0.668 ± 0.031	0.754 ± 0.010	1.129 ± 0.038	11.362 ± 2.985	26 ± 1.324
ODT7	0.621 ± 0.015	0.734 ± 0.025	1.165 ± 0.034	11.654 ± 2.364	29 ± 1.265
ODT8	0.581 ± 0.013	0.639 ± 0.016	1.148 ± 0.027	12.185 ± 2.139	24 ± 2.654
ODT9	0.565 ± 0.015	0.695 ± 0.011	1.139 ± 0.023	12.952 ± 1.912	29 ± 1.632

**Table 5:** Parameters of ODTs.

Parameters formulations	Thickness (mm)	Weight (mg)	Hardness (kg/cm^2^)	DT (s)	WT (s)	Friability (%)
ODT1	3.175 ± 0.014	151.8 ± 3.551	3.1 ± 0.152	32	40	0.69
ODT2	3.042 ± 0.026	150.7 ± 3.632	3.0 ± 0.096	27	35	0.66
ODT3	3.143 ± 0.034	149.2 ± 2.427	2.9 ± 0.126	21	31	0.62
ODT4	3.025 ± 0.004	147.8 ± 3.321	2.8 ± 0.134	20	30	0.59
ODT5	3.094 ± 0.037	151.1 ± 2.731	2.8 ± 0.157	17	25	0.56
ODT6	3.042 ± 0.029	146.5 ± 3.654	2.7 ± 0.095	15	24	0.55
ODT7	3.163 ± 0.034	149.8 ± 2.427	2.9 ± 0.126	14	23	0.54
ODT8	3.175 ± 0.024	150.8 ± 3.251	3.0 ± 0.153	13	22	0.52
ODT9	3.114 ± 0.047	149.1 ± 2.631	2.8 ± 0.167	11	21	0.55

**Table 6:** This table shows goals and solution of optimized tablet as suggested by the software.

Constraints					
Name	Goal	Lower limit		Upper limit	
SSG	In range	−1		1	
Crospovidone	In range	−1		1	
DT (s)	Target = 15	11		32	
WT (s)	In range	21		40	
Friability (%)	Minimize	0.52		0.69	

Solution					
SSG (*X*_1_)	Crospovidone (*X*_2_)	DT (s)	WT	FB (%)	Desirability

−0.36	0.56	15	23.58	0.54	0.942

**Table 7:** Summary of results of regression analysis.

	*b*_*o*_	*b*_1_	*b*_2_	*b*_12_	*b*_11_	*b*_22_
Response (disintegration time)/coefficients						
FM	17.44	−3.17	−7.00	2.00	−0.17	2.33

Response (wetting time)/coefficients						
FM	25.78	−2.83	−6.67	1.75	0.83	2.33

Response (% friability)/coefficients						
FM	0.56	−0.017	−0.060	0.020	1.00	0.030

## References

[1] Mohapatra A., Parikh R. K., Gohel M. C. (2008). Formulation, development and evaluation of patient friendly dosage forms of metformin. Part-I. Orally disintegrating tablets. *Asian Journal of Pharmaceutics*.

[2] Hirani J. J., Rathod D. A., Vadalia K. R. (2009). Orally disintegrating tablets: a review. *Tropical Journal of Pharmaceutical Research*.

[3] Xu J., Bovet L. L., Zhao K. (2008). Taste masking microspheres for orally disintegrating tablets. *International Journal of Pharmaceutics*.

[4] Habib W., Khankari R., Hontz J. (2000). Fast-dissolve drug delivery systems. *Critical Reviews in Therapeutic Drug Carrier Systems*.

[5] Sastry S. V., Nyshadham J. R., Fix J. A. (2000). Recent technological advances in oral drug delivery—a review. *Pharmaceutical Science and Technology Today*.

[6] Fu Y., Yang S., Jeong S. H., Kimura S., Park K. (2004). Orally fast disintegrating tablets: developments, technologies, taste-masking and clinical studies. *Critical Reviews in Therapeutic Drug Carrier Systems*.

[7] Bandari S., Mittapalli R. K., Gannu R., Rao Y. M. (2008). Orodispersible tablets: an overview. *Asian Journal of Pharmaceutics*.

[8] Mali Sandip L., Nighute Ashok B., Vivek D., Gonjari Indrajeet D., Bhise Satish B. (2010). Microcrystals: for improvement of solubility and dissolution rate of Lamotrigine. *International Journal of Pharmaceutical Sciences*.

[9] Shah H. J., Subbaiah G., Patel D. M., Patel C. N. (2009). *In vitro*-*in vivo* correlation of modified release dosage form of lamotrigine. *Biopharmaceutics & Drug Disposition*.

[10] Amrutkar P. P., Patil S. B., Todarwal A. N., Wagh M. A., Kothawade P. D., Surawase R. K. (2010). Design and evaluation of taste masked chewable dispersible tablet of lamotrigine by melt granulation. *International Journal of Drug Delivery*.

[11] Birhade S. T., Bankar V. H., Gaikwad P. D., Pawar S. P. (2010). Preparation and evaluation of cyclodextrin based binary systems for taste masking. *International Journal of Pharmaceutical Research and Development*.

[12] Goel H., Rai P., Rana V., Tiwary A. K. (2008). Orally disintegrating systems: innovations in formulation and technology. *Recent Patents on Drug Delivery and Formulation*.

[13] Garg R., Gupta G. D. (2009). Preparation and evaluation of gastroretentive floating tablets of acyclovir. *Current Drug Delivery*.

[14] Modi A., Tayade P. (2006). Enhancement of dissolution profile by solid dispersion (kneading) technique. *AAPS PharmSciTech*.

[15] Shid R. L., Dhole S. N., Kulkarni N., Shid S. L. (2014). Formulation and evaluation of nanosuspension delivery system for simvastatin. *International Journal of Pharmaceutical Sciences and Nanotechnology*.

[16] Shan-Yang L., Yuh-Horng K. (1989). Solid particulates of drug-*β*-cyclodextrin inclusion complexes directly prepared by a spray-drying technique. *International Journal of Pharmaceutics*.

[17] Krishanaiah Y. S. R., Latha K., Nageswara Rao L., Karthikeyan R. S., Bhaskar P., Satyanarayana V. (2003). Development of colon targeted oral guar gum matrix tablets of Albendazole for the treatment of helminthiasis. *Indian Journal of Pharmaceutical Sciences*.

[18] Her Majesty's Stationary Office (2000). *British Pharmacopoeia*.

[19] Setty C. M., Radhika M., Gupta V. R. M., Reddy M. V. R., Jithan A. V. (2011). Effect of tablet processing and formulation factors on disintegration and dissolution of aceclofenac tablets. *International Journal of Pharmaceutical Sciences and Nanotechnology*.

